# Ion Concentration Polarization by Bifurcated Current Path

**DOI:** 10.1038/s41598-017-04646-0

**Published:** 2017-07-11

**Authors:** Junsuk Kim, Inhee Cho, Hyomin Lee, Sung Jae Kim

**Affiliations:** 1 Department of Electrical and Computer Engineering, Seoul National University, Seoul, 08826 Republic of Korea; 2 Big Data Institute, Seoul National University, Seoul, 08826 Republic of Korea; 3 Inter-university Semiconductor Research Center, Seoul National University, Seoul, 08826 Republic of Korea

## Abstract

Ion concentration polarization (ICP) is a fundamental electrokinetic process that occurs near a perm-selective membrane under dc bias. Overall process highly depends on the current transportation mechanisms such as electro-convection, surface conduction and diffusioosmosis and the fundamental characteristics can be significantly altered by external parameters, once the permselectivity was fixed. In this work, a new ICP device with a bifurcated current path as for the enhancement of the surface conduction was fabricated using a polymeric nanoporous material. It was protruded to the middle of a microchannel, while the material was exactly aligned at the interface between two microchannels in a conventional ICP device. Rigorous experiments revealed out that the propagation of ICP layer was initiated from the different locations of the protruded membrane according to the dominant current path which was determined by a bulk electrolyte concentration. Since the enhancement of surface conduction maintained the stability of ICP process, a strong electrokinetic flow associated with the amplified electric field inside ICP layer was significantly suppressed over the protruded membrane even at condensed limit. As a practical example of utilizing the protruded device, we successfully demonstrated a non-destructive micro/nanofluidic preconcentrator of fragile cellular species (*i*.*e*. red blood cells).

## Introduction

Recent nanoscale electrokinetic researches have focused on understanding a novel ion transportation through nanoporous membrane^1, 2^ or nanochannel system^3–7^, since it forms essential basis for developing various engineering applications such as nanofluidic diodes^8–12^, energy harvesting device^13, 14^, (bio-)molecular preconcentrator^15–20^ and electro-desalinator^21–25^. Among the interesting transport phenomena, ion concentration polarization (ICP) has drawn significant attentions because it describes the motion of ions under an electric field, which resembles a phenomenon occurs inside a body or a cell^26, 27^. The essential mechanism of ICP is that only counter-ions can pass through a charged nanoporous membrane under dc bias^28–30^. This results to the imbalance of ion concentrations at anodic and cathodic side of the membrane. The regions with low concentration at anodic side and high concentration at cathodic side are called an ion depletion zone and an ion enrichment zone, respectively^7, 28^. The fingerprinting evidence of these zones has been reported as the appearance of the Ohmic-limiting-overlimiting current regimes^28^. Especially, the source of overlimiting current, which was unable to be described by a conventional diffusion-drift theory^26^, has been debated for a decade to include the dominance regime of extended space charge layer^31^, surface conduction^1, 5^, electro-diffusio-convection^2, 28, 32^ and electroosmotic instability^33, 34^. Once the porous material has the permselectivity, the deterministic factors of distinguishing the regimes are external parameters such as the characteristic dimensions of bulk environment, the surface charges of building substrates and the bulk electrolyte concentrations^5^. If the externals become larger dimensions, lower surface charge and higher bulk concentration, the ICP phenomena enter into the unstable regime^5^. Consequently, most of engineering ICP applications to concentrate bio-sample and to desalinate saline water would have been suffered from the unintended instability^23, 35, 36^ since they utilized higher concentration than physiological fluid’s.

To avoid this nuisance, the underlying physics should be carefully investigated. The instability and the chaotic motions of the ion depletion zone are caused by the thermal fluctuation of the extended space charge layer and the amplified electric field inside the ion depletion zone with the absence of the surface conduction and the electro-diffusio-convection^37^. Thus, the instability would be mitigated by the enhancement of the surface conduction or electro-diffusio-convection. In this sense, we artificially enhanced the surface conduction by coating a highly conductive polymer inside the microchannel. Since Nafion of the nanoporous membrane has high electrical conductance by itself, we let Nafion protrude along the microchannel so that there was an additional current path (or a bifurcated path) through the protruded part of Nafion, while Nafion was exactly aligned at the interface between two microchannels in the conventional ICP device. Lithographically fabricated nanochannels were unable to achieve this surface conduction enhancement because of a lower surface charge of substrates than one of Nafion (*e*.*g*. silicon, glass and PDMS ~ 10–50 mC/m^2^ and Nafion ~ 200–600 mC/m^2^)^38^. Rigorous characterization of this protruded device by both experiments and numerical simulations would be given here and, finally, a non-destructive preconcentration of fragile cells (red blood cell (RBC)) that required a stable ICP layer at high concentration and minimizing the shear stress due to strong electro-convection was successfully demonstrated.

## Materials and Methods

### Experimental setups

**Figure 1:**
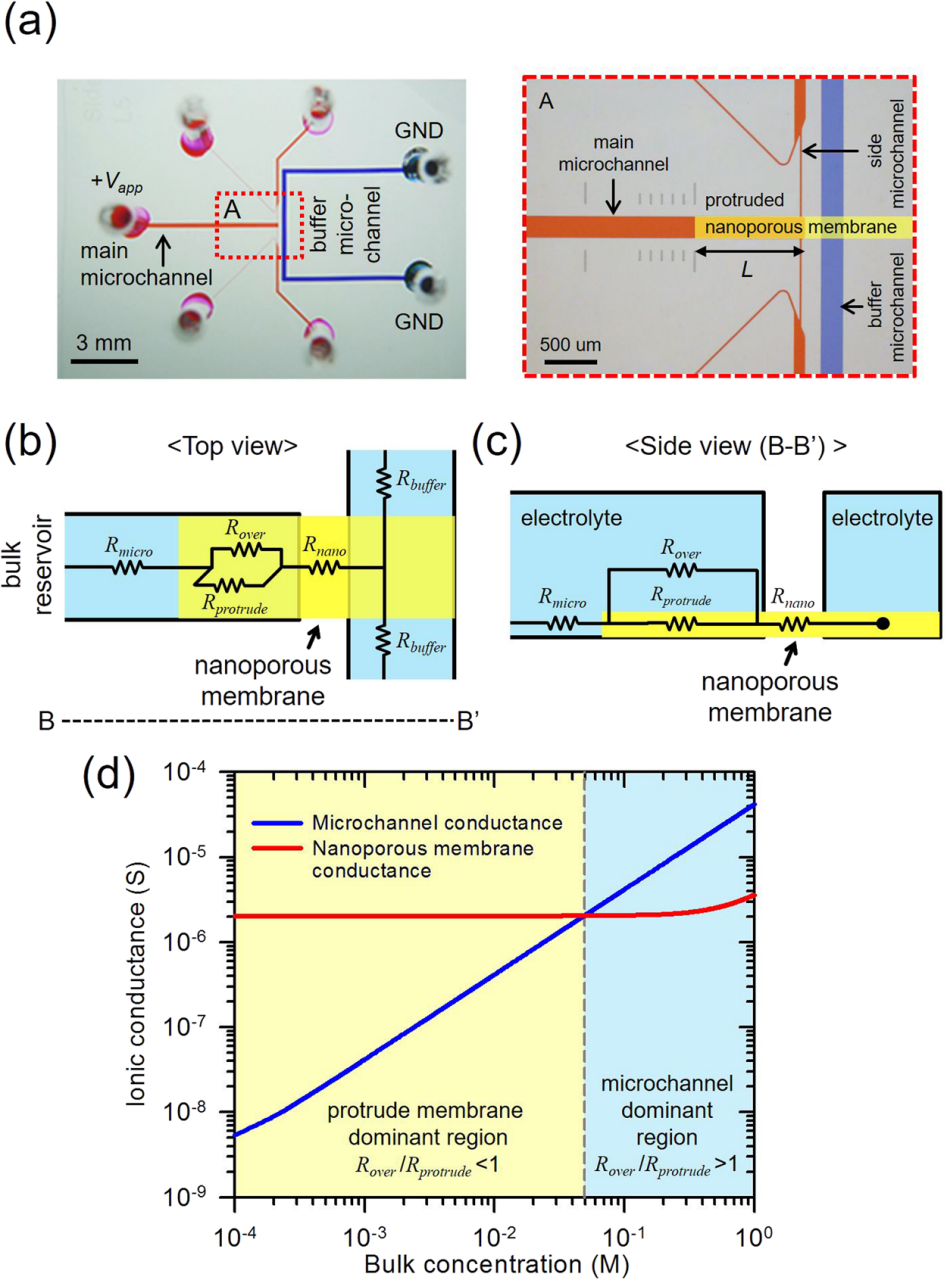
(**a**) Snapshot and microscopic view ((A) in red box) of the fabricated non-destructive cellular preconcentrator. *L* was the length of protruded nanoporous membrane from the main microchannel. Schematics of (**b**) top and (**c**) side view of the proposed devices with equivalent electrical resistors (not to scale). (**d**) Calculated ionic conductance of microchannel and nanoporous membrane.

The design of the microchannel layout was similar to those devices that we have previously investigated as shown in Fig. 1(a) ^39^. On the main microchannel, we implemented the air valve structures which allowed the main microchannel to become a dead-end microchannel so that one can conduct the experiment easily, while obtaining the exact electrokinetic response^39^. The dimensions of the microchannel network were as follows: the main- and buffer-microchannel: 200 μm width × 15 μm depth, the side-microchannel of narrow region: 15 μm width × 15 μm depth, and the side-microchannel of wide region: 100 μm width × 15 μm depth. The device were fabricated by conventional PDMS fabrication steps^40^. The Nafion (Sigma-Aldrich, USA) as a nanoporous membrane with high electrical conductivity, was patterned on the glass substrate using surface patterning method^41^. Briefly, Nafion was patterned by a simple straight microchannel (200 μm width × 50 μm depth) on a glass slide. Then, PDMS block having a microchannel network was irreversibly bonded by plasma bonder (CuteMP, Femto Science, Korea) to a designated position on top of the Nafion-patterned glass. Note that the protruded portion of Nafion membrane (denoted as *L* in the Fig. 1(a)) should be aligned within the main microchannel under microscopic observations.

For visualization experiments, a mixture of KCl solution (Sigma-Aldrich, USA) at a concentration ranging from 0.1 mM to 1 M with Alexa Flour 488 (1 μM, Invitrogen, USA) as a fluorescent tracer were injected to both microchannels. pH of the KCl solution was measured to be around 5.6. Voltage was applied from the main microchannel reservoir via Ag/AgCl electrodes (a source measure unit, Keithley 236, USA) to the buffer microchannel reservoirs, forming the ion depletion zone at the main microchannel. The propagations of ICP layer were imaged by an inverted fluorescent microscope (IX53, Olympus) and CellSens program.

For current−time response measurements, a constant external voltage of 50 V were applied and the current values were automatically recorded at every 0.25 seconds by Labview program. 1 × phosphate buffer saline (PBS) was chosen as a test solution which is a buffer solution commonly used in biological research. Both visualization and I-t measurements were conducted at least 5 times with 5 different devices to ensure repeatability and reliability.

For non-destructive preconcentration experiments, human whole blood, 1 × PBS and 500 mM EDTA as the anticoagulant, was mixed at a volume ratio of 1:50:0.5 as a target sample.

### Numerical methods: Three-layers model

**Figure 2:**
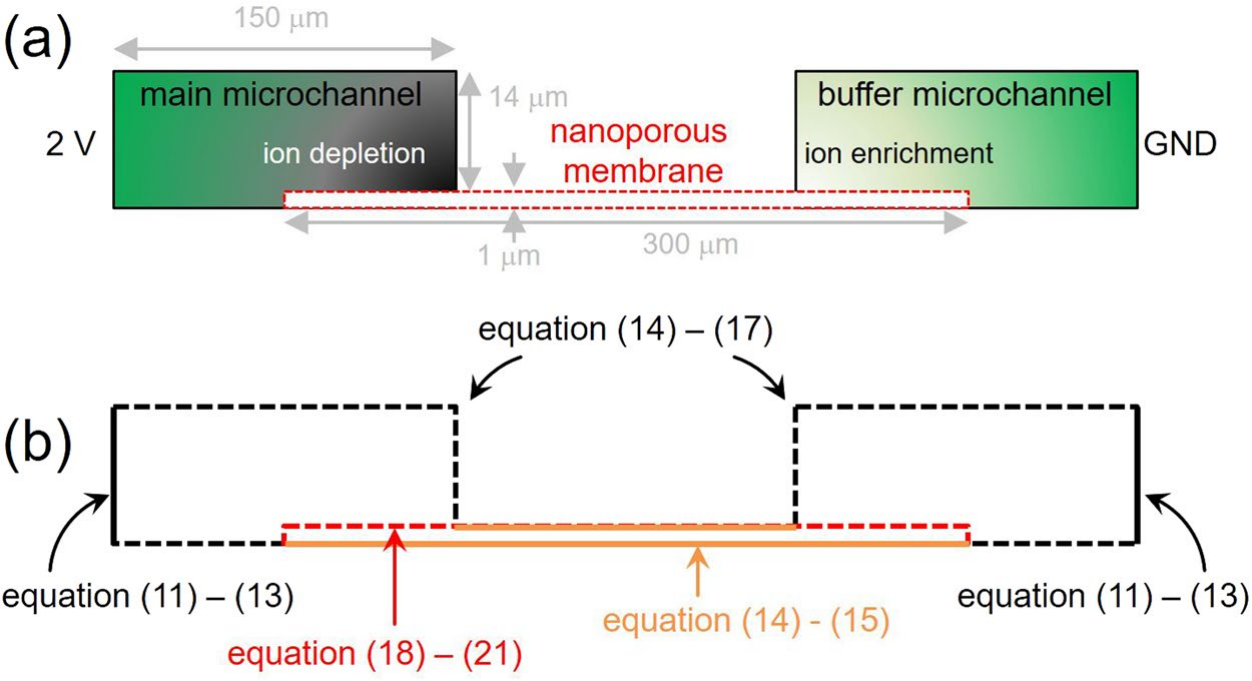
(**a**) Schematic diagram of numerical domain for the protruded nanoporous membrane system. The size of the buffer microchannel was identical to main microchannel. The three-layer means “microchannel”-“membrane”-“microchannel”. Note that the figure was not to scale. (**b**) Schematic diagram of summarized boundary conditions. The boundaries were divided into four types which were represented as black solid, black dash, orange solid and red dash lines.

In order to describe ICP phenomenon above the protruded nanoporous membrane, we established a numerical model which was based on the three-layer model suggested by Rubinstein and Zaltzman^42^ for saving computational costs^43, 44^. The computational domain was depicted in Fig. 2(a). Since we chose a shorter length scale than one of the actual experimental device to enhance the numerical stability and reduce the computational cost, the spatiotemporal ICP layer dynamics on the protruded membrane were qualitatively described. Nevertheless, the qualitative results would provide invaluable physical insights to explain the experimental observations because the ion concentration, the electric field and the flow field inside the actual device were unable to be measured directly.

The main and buffer microchannel had following electroneutral condition if we neglect the thickness of electrical double layer on microchannel walls and membrane surfaces.

1 $${c}_{+}={c}_{-}=c$$

where *c*_+_ is the cation concentration, *c*_−_ is the anion concentration and *c* is the local ionic strength which is defined as *c* = (*c*_+_ + *c*_−_)/2 for 1:1 electrolyte. However, the electroneutral condition inside the cation-selective membrane was

2 $${c}_{+}-{c}_{-}-N=0$$

where *N* is the fixed charge concentration of the membrane. For convenience, we set up the positive *N* for the cation-selective membrane. Using the definition of the local ionic strength, the cation and anion concentrations inside the membrane were represented as

3 $${c}_{+}=c+\frac{N}{2}$$

and

4 $${c}_{-}=c-\frac{N}{2}.$$

Conventionally, using the Equation (1), the Nernst-Planck equations to describe the ionic transportation inside the main and buffer microchannel were decoupled into two equations: the diffusion-convection equations and current conservation equations^39, 45, 46^ which are

5 $$\frac{\partial c}{\partial t}=-\nabla \cdot (-D\nabla c+c{\bf{u}})$$

and

6 $$0=-\nabla \cdot (-\frac{2{F}^{2}D}{RT}c\nabla \psi )$$

where *t* is the time, *D* is the diffusivity, **u** is the flow field, *F* is the Faraday constant, *R* is the gas constant, *T* is the absolute temperature, *ψ* is the electric potential. Using the same procedures and electroneutral conditions (Equations (3) and (4)), the Nernst-Planck equations for the nanoporous membrane became

7 $$\frac{\partial c}{\partial t}=-\nabla \cdot (-D\nabla c-\frac{FD}{RT}\frac{N}{2}\nabla \psi )$$

and

8 $$0=-\nabla \cdot (-\frac{2{F}^{2}D}{RT}c\nabla \psi )$$

under theoretical assumptions of (1) low permeability (**u = **0 in the nanoporous membrane) and (2) homogeneous membrane property (*N* = constant for entire membrane). See the detailed derivation in supporting information. In summary, the ionic transportation in the main and buffer microchannel was governed by Equations (5) and (6) while the ionic transportation in the nanoporous membrane was governed by Equations (7) and (8). The flow field was governed by the Stokes equations under thin electrical double layer approximation

9 $$\rho \frac{\partial {\bf{u}}}{\partial t}=-\nabla p+\eta {\nabla }^{2}{\bf{u}}$$

and the continuity equation

10 $$\nabla \cdot {\bf{u}}=0$$

for the incompressible fluid. In the above equations, *ρ* is the density of water, *p* is the pressure, *η* is the dynamic viscosity of water. The Equations (9) and (10) were applied only on the main and buffer microchannel since the nanoporous membrane in this work has low water permeability.

With the above governing equations, the following boundary conditions were used. On the bulk interface,

11 $$c={c}_{0}$$

12 $$\psi ={V}_{specific}$$

and

13 $$p=0$$

where *c*_0_ is the bulk concentration and *V*_*specific*_ means the specific value of the electric potential whose value was set to be 2 V on the left bulk interface and 0 V on the right interface. On the microchannel walls,

14 $${\bf{n}}\cdot \nabla c=0$$

15 $${\bf{n}}\cdot \nabla \psi =0$$

16 $${\bf{n}}\cdot {\bf{u}}=0$$

and

17 $${\bf{t}}\cdot {\bf{u}}=\frac{\varepsilon {\zeta }_{w}}{\eta }{\bf{t}}\cdot \nabla \psi $$

where **n** is the outward normal vector and **t** is the tangential vector, *ε* is the electrical permittivity of water and *ζ*_*w*_ is the zeta potential of the microchannel walls^47^. Equations (14), (15) and (16) implied the conditions of no-penetration of ionic flux and ionic current. Equation (17) is the Smoluchowski slip velocity under the thin electrical double layer approximation. Lastly, on the membrane surfaces,

18 $${{\bf{n}}\cdot \nabla c|}_{microchannel}={{\bf{n}}\cdot \nabla c|}_{membrane}$$

19 $${{\bf{n}}\cdot \nabla \psi |}_{microchannel}={{\bf{n}}\cdot \nabla \psi |}_{membrane}$$

20 $${\bf{n}}\cdot {\bf{u}}=0$$

and

21 $${\bf{t}}\cdot {\bf{u}}=\frac{\varepsilon {\zeta }_{n}}{\eta }{\bf{t}}\cdot \nabla \psi $$

where *ζ*_*n*_ is the zeta potential or the surface potential of the nanoporous membrane which was obtained by the experiment in Supporting information. Equations (18) and (19) implied that the ionic flux and the ionic current are continuous at the interface of microchannel and the nanoporous membrane. The *ζ*_*n*_ can be evaluated by the Donnan equilibrium^48^ which was

22 $${\zeta }_{n}=-\frac{RT}{F}\,\mathrm{ln}\,\frac{\sqrt{4{{c}_{0}}^{2}+{N}^{2}}+N}{2{c}_{0}}$$

for the cation-selective membrane. The above boundary conditions were summarized in Fig. 2(b). The governing equations with the boundary conditions were simulated with commercial FEM software (COMSOL multiphysics 4.4).

## Results and Discussion

### The characterization of bifurcated current path with equivalent circuit

Microscopic snapshot of the micro/nanofluidic device having the protruded nanoporous membrane was shown in Fig. 1(a). The length of the protruded nanoporous membrane was denoted by *L*. While conventional nanoporous membrane was positioned right at the end of the microchannel substrate, *i*.*e*. *L* = 0 um, the nanoporous membrane was intentionally protruded from the end of the microchannel substrate in this device. For example, the devices of *L* = 0 um, 1000 um, 3000 um or 6500 um were fabricated depending on the experimental needs. By installing the protruded membrane, one can expect that the path of ionic flux was bifurcated as described by the equivalent circuit model. Schematics of top and side view of the proposed devices with equivalent electrical resistors were shown in Fig. 1(b) and (c), respectively. *R*_*micro*_ and *R*_*buffer*_ represented the resistors of the main and buffer microchannel. *R*_*nano*_ referred the resistor of the nanoporous membrane between the main and buffer microchannel. While *R*_*over*_ and *R*_*protrude*_ had been ignored in a conventional nanofluidic device, they appeared in this device since the highly conducting membrane was protruded, leading to the bifurcated current path. Since ionic current tended to pass through the lower resistor, the ratio of *R*_*over*_ to *R*_*protrude*_ played the significant role to determine the effective current path and the location where ICP was initiated. Here, *R*_*protrude*_ was fixed by the material property of the membrane and *R*_*over*_ was inversely proportional to the electrolyte concentration inside the microchannel so that the current path highly depended on the electrolyte concentration.

In order to characterize the dependency, two microfluidic devices were fabricated for the comparison of the conductance between a nanoporous membrane and the electrolyte inside a microchannel. The first device was a simple straight microchannel (SI Fig. 1(a)) and the second device was composed of a patterned nanoporous membrane at the bottom of the same simple straight microchannel (SI Fig. 1(b)). Detailed dimensions of the devices and the measurement method were described in supporting information. The ionic conductance of the first device, which only had a microchannel part, was proportional to the bulk concentration (SI Fig. 1(c)), while the ionic conductance of the second device which had a parallel connection of the microchannel and the nanoporous membrane, formed a plateau below a threshold concentration (SI Fig. 1(d)).

The appearance of plateau is attributed to electric double layer (EDL) overlap phenomenon which becomes severe as the bulk concentration decreases. Since fixed amount of counter-ions existed inside the nanoporous membrane with the severe EDL overlap, the ionic conductance of the nanoporous membrane was independent from the bulk concentration^30, 49^.

This estimation was applied to the protrude membrane device to identify the effective path of ionic flux passing through either the protrude nanoporous membrane or the microchannel. The calculated ionic conductance were plotted in Fig. 1(d) as a function of the electrolyte concentration ranging from 1 × 10^−4^ to 1 M. As a result, the dominant path of ionic flux was bifurcated near 50 mM in the device. In other words, the current dominantly conducted through the protrude membrane at the concentration lower than 50 mM and *vice versa*.

### The propagation of ICP layer upon bifurcated current path

**Figure 3:**
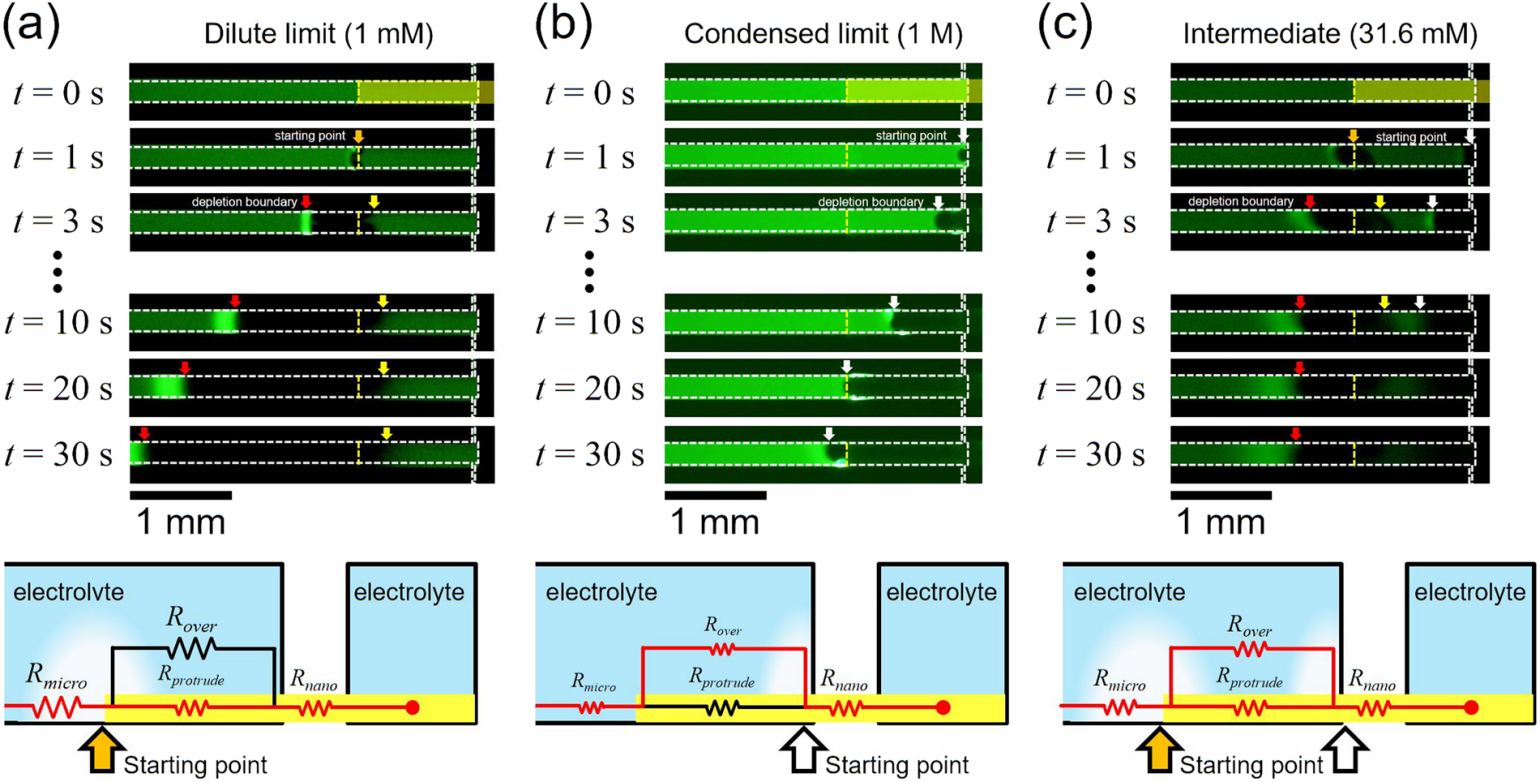
Time-revolving snapshots of propagating ion depletion zone (or ICP layer) at each concentrations with the applied voltage of 50 V; (**a**) Dilute limit (1 mM), (**b**) condensed limit (1 M) and (**c**) intermediate (31.6 mM). See supporting video for each figures. The schematic connections with the equivalent circuit were also given below each figures (not to scale). Red line indicated the dominant current paths.

The bifurcated path was confirmed by the visualization of ICP layer formation. The experiments were conducted with the protruded nanoporous membrane of *L* = 1000 um. Three different electrolyte concentrations were selected as representative values based on the data in Fig. 1(d); (i) dilute limit: 1 mM for nanoporous membrane dominant regime (*R*_*over*_/*R*_*protrude*_ > 1), (ii) condensed limit:1000 mM for microchannel dominant regime (*R*_*over*_/*R*_*protrude*_ < 1), and (iii) 31.6 mM for intermediate regime (*R*_*over*_/*R*_*protrude*_ ≈ 1). The time-revolving snapshots of propagating ion depletion zone (or ICP layer) at each concentrations were shown in Fig. 3(a–c). The schematic connections using the equivalent circuit were also given below each figures. Red line indicated the dominant current paths.

**Figure 4:**
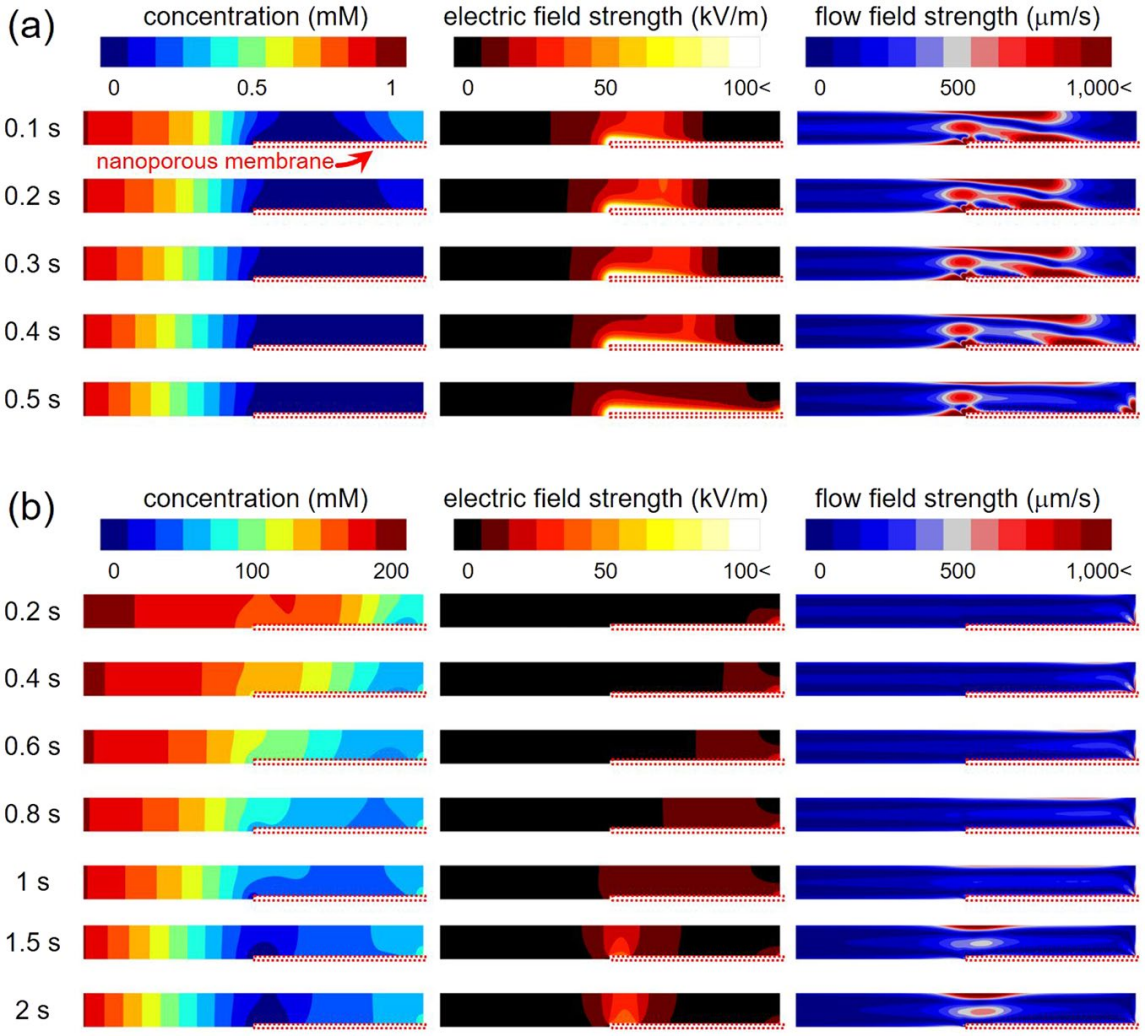
The numerically simulated spatiotemporal formation of the ion depletion zone in the case of (**a**) dilute limit and (**b**) condensed limit. The membrane charge concentration (*N*) was fixed at 100 mM. In each case, the bulk concentration was 1 mM and 200 mM respectively.

At dilute limit (1 mM), the propagation of the ion depletion zone started at the end of the protruded nanoporous membrane (Fig. 3(a)) and expanded toward both directions. See the supporting video. The propagation toward anodic side (red arrow) was similar to the conventional propagation, where the amplified electric field and chaotic electro-convection mixed the electrolytes^50^. However, a reverse propagation toward the cathodic side (yellow arrow) was also observed. This propagation was comparatively slow and the boundary moved nearly zero velocity. The main factor for the slow propagation was due to near zero electric field above the protrude membrane as shown in Fig. 4(a) which was the simulation results. Because the conductance of nanoporous membrane was much higher than that of microchannel in the dilute limit, the preferential path of the ionic current should be through the protruded membrane, *i*.*e*. no strong electro-convection over the membrane. As a result, the diffusion was the only driving force for the propagation (yellow arrow), leading to the blurred boundary, while the propagation due to a strong electro-convection (red arrow) had a sharp boundary.

On the contrary, the propagation started from the end of the microchannel (white arrow) and expanded until the zone met the end of protruded membrane in the case of condensed limit (1 M) (Fig. 3(b)). See the supporting video. Typically, the propagation characteristics of the ion depletion zone in highly concentrated limit such as over 100 mM can be explained as unstable, slow propagation, and short ion depletion zone length^23, 36^. Noteworthy, stable and lengthy propagation of the ion depletion zone was observed over this protruded membrane as well in Fig. 4(b), because the current path was formed through the membrane. However, once the depletion zone escaped the membrane (*t* > 20 sec in Fig. 3(b)), strong electro-convection reappeared, since the concentration above the protruded membrane became diluted and behaved as the conventional propagation with strong vortex. This scenario was confirmed by the numerical simulation as well.

In order to explain the distinguishable formation of ICP layer above the protruded nanoporous membrane, we established a numerical model which was based on the three-layer model suggested by Rubinstein and Zaltzman^42^ as described in Numerical Methods section. Figure 4(a) showed the results of the dilute limit case. If the bulk concentration is lower than the fixed charge concentration of the membrane (the fixed charge concentration (*N*) was set to be 100 mM in simulations), the most of the ionic current would pass through the protruded part (*R*_*protrude*_ in Fig. 3) instead of the microchannel part over the membrane (*R*_*over*_ in Fig. 3). Thus, the ion depletion zone was initiated at the end of the membrane as shown in the first column of Fig. 4(a). As the ion depletion zone was formed, the amplified electric field and strong electro-convection were generated (the second and third column of Fig. 4(a)). The high electric field on the protruded membrane (white region) reflected the high potential gradient between the protruded membrane and the electrolyte solution on the membrane due to a significant conductance difference. Note that the concentration shown in the first column was the ionic concentration so that the experimental visualization by fluorescent molecules in Fig. 3(a) was qualitatively agreed. On the other hand, in the condensed limit which means the bulk concentration is higher than the fixed charge concentration, the ionic current predominantly pass through the upper part of the membrane (*R*_*over*_ in Fig. 3) so that the initiating point of the ion depletion zone was shifted toward the end of microchannel comparing to the case of dilute limit as shown in the first column of Fig. 4(b). Although the ion depletion zone was started at the end of the microchannel, the minimum concentration region (dark blue colored area) was eventually positioned at the end of the membrane. As shown in the second and the third column of Fig. 4(b), the strength of electric field and flow field was greatly smaller than the case of dilute limit. However, the strength of the electro-hydrodynamic fields increased to the similar level of the dilute limit case when the ion depletion zone was fully developed (after 2 sec in the simulations).

At an intermediate concentration (31.6 mM), the propagation started at both ends of protruded membrane and the microchannel. (Fig. 3(c)), showing like a merged picture of the dilute limit and the condensed limit. See the supporting video. These phenomena can also be explained by the resistance ratio between microchannel and nanoporous membrane (*R*_*over*_ and *R*_*protrude*_ in Fig. 1(c)). In the case of *R*_*over*_/*R*_*protrude*_ ≈ 1, the current path would be formed every points along the membrane, but the electric field at the ends of protruded membrane and the microchannel would be amplified due to geometric focusing (or edge) effects so that two current paths were competing to induce two ion depletion zones. Note that numerical simulation was not available for this case due to singular condition of *R*_*over*_/*R*_*protrude*_ ≈ 1.

### I-t responses upon bifurcated current path

To explore the mechanisms of how the current level changes as the ion depletion zone propagates, I-t responses were measured at 0.01 × PBS media (~1.5 mM; dilute limit) and 1 × PBS media (~150 mM; condensed limit) with the various protruded length. We selected PBS since it was common biological buffer solution and the concentration of 1 × PBS (~150 mM) was laid in the condensed limit.

**Figure 5:**
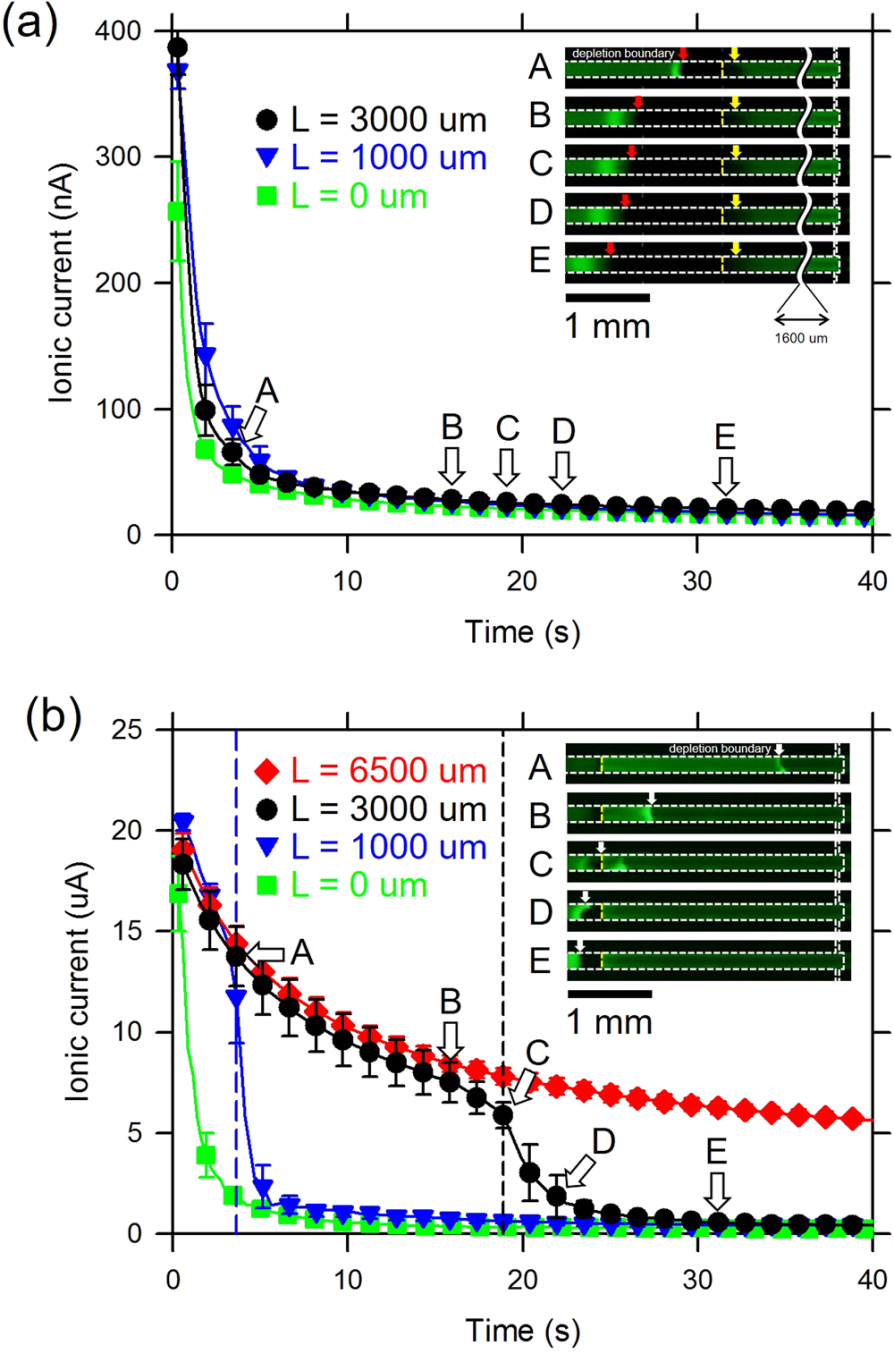
Current-time responses of (**a**) 0.01 × PBS media (~1.5 mM; dilute limit) and (**b**) 1 × PBS media (~150 mM; condensed limit) with the various protruded length. Each figures had an inset of microscopic views in the case of *L* = 3000 um. Capital letters indicated the time at which the snapshot were taken.

In the case of dilute limit, the depletion zone started from the end of protruded membrane so that the current dropped immediately and decayed exponentially for all protruded length as shown in Fig. 5(a). The device in the inset had *L* = 3000 um. This was because the remaining portion of solution over the membrane merely affected on the current path. Thus, all of data converged into the case of non-protrude membrane (*L* = 0 um).

On the contrary, at the condensed limit, the ion depletion zone started at the end of the microchannel quickly propagating toward the anodic side (A ~ C in the inset of Fig. 5(b)) and the propagation was slowed down once the boundary reached the end of membrane (D ~ E in the inset of Fig. 5(b)). The device in the inset had *L* = 3000 um. This observation taught us that the propagation was divided into two stages. This was also confirmed by the I-t drops as well in Fig. 5(b). See the line of *L* = 3000 um as a representative example. The first current drop had low rate while the ion deletion zone boundary moved over the protruded membrane (A ~ B in Fig. 5(b)). The second stage had the high rate of current drop, where the boundary was located outside the protruded membrane (D ~ E in Fig. 5(b)). In the first stage, even though the ion depletion zone length increased dramatically (0 um to 3000 um), the current only decreased to one third of its initial value. In the second stage, there was a small increment of the ion depletion zone length (3000 um to 3350 um) but the current decreased dramatically to one fiftieth of its initial value. Conventionally current decreased rapidly with the formation of the ion depletion zone like the behavior in the second stage or the behavior of *L* = 0 um case. This was because of the rapid concentration drop inside the depletion zone, resulting extremely high electrical resistance and the amplified electric field inside the depletion zone^50^. In the second stage, the current was passing mostly through the protruded membrane even in this condensed limit because the electrolyte concentration over the membrane was significantly depleted. In summary, since the conductance of the protruded membrane was fixed in the first and second stage, the main current path would be changed from the electrolyte solution over the membrane in the first stage to the membrane in the second stage. In the first stage where the current decreased slowly, the effective conductance of the ion depletion zone maintained higher than that of conventional depletion zone with the aid of the constant conductance of the protruded membrane. That is to say, once the depletion zone was initiated (*i*.*e*. *R*_*over*_ started to increase), the most of current started to conduct through *R*_*protrude*_, since *R*_*over*_ and *R*_*protrude*_ were connected in parallel. Therefore, the magnitude of local electric field over the protrude membrane in the first stage was significantly less than that in the conventional device, leading to the minimal magnitude of amplified electrokinetic flow inside the zone and its associated shear stress near the zone. Also see the numerical results in Fig. 4.

In the following section, a non-destructive cellular preconcentrator was demonstrated with extremely long protrude membrane for maximizing the first stage.

### Non-destructive Cellular Preconcentrator

ICP had been utilized as an efficient biomolecular preconcentration mechanism^15^. Unlike a preconcentration mechanism using a free-flow concept such as isotachophoresis^51^ and field amplified stacking^52^, the amplification ratio of ICP preconcentrator would be up to million-fold since the target molecules was supplied from a reservoir of nearly infinite volume compared with the volume of microchannel^53^. While various (bio-)molecules such as fluorescent dyes^16, 54^, DNAs and aptamers^55^, *etc*. were successfully preconcentrated with noticeable factor, there are intrinsic limitations to be solved. First, most of the device using ICP phenomena has been operated at low bulk concentration which can cause the modification or destruction of the biosamples due to osmotic pressure in the case of cellular species^56, 57^. The main reason of using a low bulk concentration was due to the instability and the chaotic motion of the ion depletion zone in high bulk concentration which hindered the stable formation of ICP layer^21, 23^. Secondly, significantly amplified electric field^50^ inside the ion depletion zone caused strong vortical flow to induce a high shear stress at the boundary of the ion depletion zone^28, 32^, destructing the target samples during ICP preconcentration process. Since most of cells were wrapped with a lipid bilayer membrane, which is only a few nanometer in thickness^58^, such cells with fragile membrane were destructed during the ICP preconcentration. Note that the shear stress of the vortex in a conventional ICP operation would increase up to 10 Pa (shear stress = water viscosity × velocity gradient = 0.001 Pa·sec × O(10) mm/sec/O(1) um)^28^. Thus, additional treatment was required for the prevention of the destruction^56^ but this process removed the original properties of the species blurring the final analysis results. Since the protrude membrane minimized the amplified electric field inside ICP layer which is directly proportional to the velocity gradient, the presenting device would be an appropriate platform for those weak cellular species.

Recently, there has been an increasing demand for a device that allows rapid blood testing anywhere, along with the growing need for point of care testing or the aim for expansion of blood testing in developing countries where the medical equipment is inadequate. Essential component of such device would be a plasma separator without loss of RBC. Since a centrifugal separation causes serious hemolysis of RBC, an efficient recovery of RBC with non-centrifugal separation would cut the volume of blood drawn to half. In this section, we would demonstrate the preconcentration of RBC without any damage.

**Figure 6:**
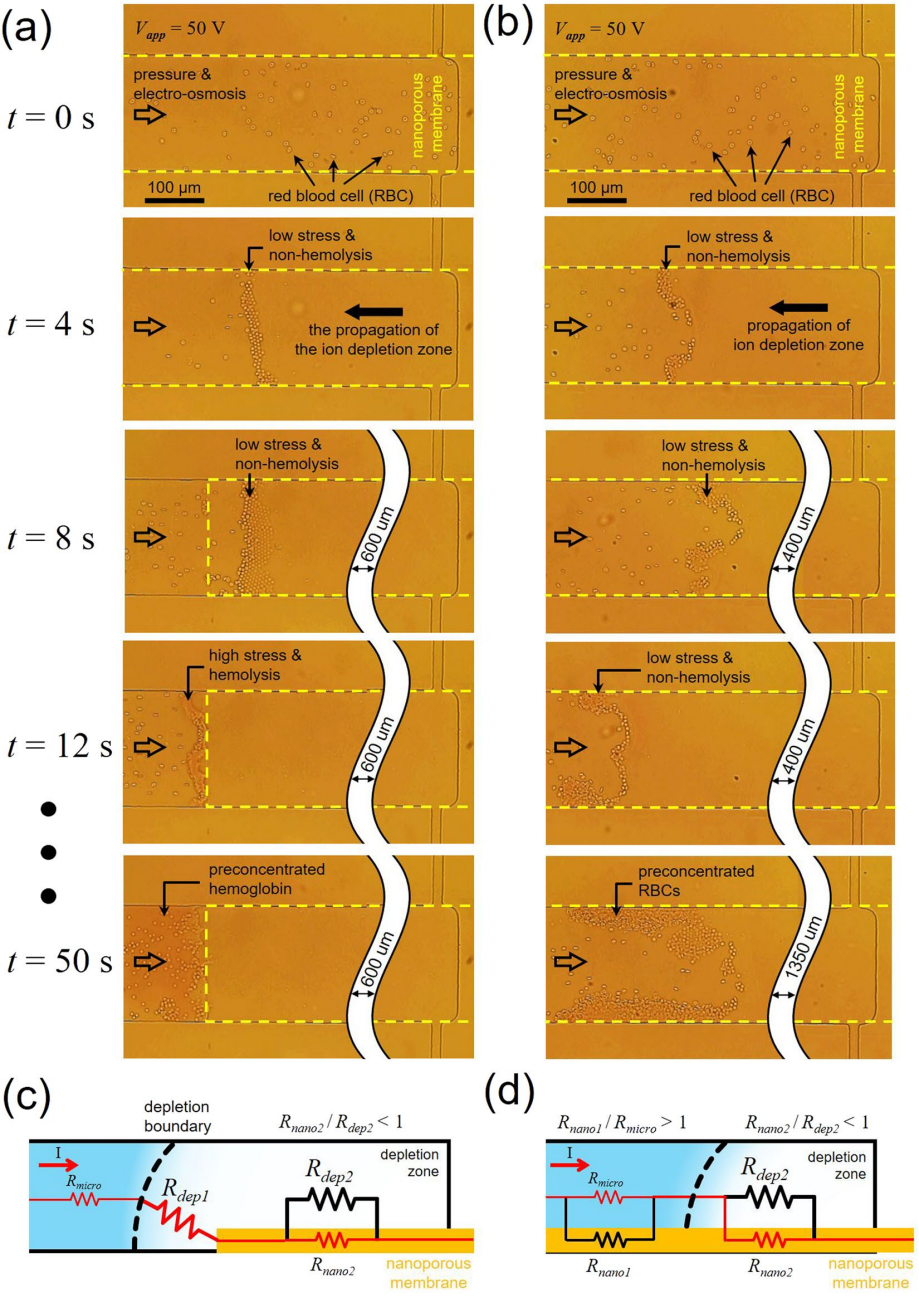
Time-revolving snapshots of RBC preconcentration with the devices of protruded membrane length (**a**) *L* = 1000 um and (**b**) *L* = 6500 um. The amplification ratio of RBCs were ~40 fold in (**b**), while most of RBCs were destroyed in (**a**). See supporting video for each figures. In terms of the current path, the schematic diagrams of developed ion depletion zone at *t* = 50 sec for each cases were depicted in (**c**) and (**d**) (not to scale).

The preconcentration of RBC out of diluted human whole blood with 1 × PBS was provided for the demonstration of the non-destructive preconcentration process. Devices with protruded membrane (*L* = 1000 um in Fig. 6(a) and *L* = 6500 um in Fig. 6(b)) were utilized in this section. The sequential snapshots of RBC preconcentration were presented for each devices. Unlike other sections which adopted closed air valve at both side-microchannels, the air valve was opened for continuous supply of sample RBCs^39^.

Alike the condensed limit in Fig. 3(b), RBC migrated toward anodic side as the ion depletion propagated (*t* = 0 ~ 8 sec) in both Fig. 6(a,b). See the supporting video. Since the ion depletion zone acted as a virtual barrier to the charged species, RBCs should rejected to enter the zone so that it should be accumulated at the boundary of the ion depletion zone. In the meantime, there was minimal amplified electric field over the protruded membrane due to the bifurcated current path. Herein there were no destruction of the blood cells in both cases. However, as the depletion zone escaped from the protruded membrane, the current path through the solution over the protruded membrane was eliminated and the strong vortical flow was formed at the end of the membrane, causing the destruction of RBCs in Fig. 6(a) from *t* = 12 sec. While it had been reported that the threshold shear stress of hemolysis was ~100 Pa at few milliseconds exposure time^59–61^, hemolysis was occurred with the vortex by ICP (shear stress = ~10 Pa) due to the combined effect of osmotic expansion of RBCs and longer exposure time. Low concentration inside ICP layer induced an osmotic pressure from outside to inside the RBSs so that they were expanded and the expansion was observed in the supporting video (The shadows of bi-concave shape would be diminished when the RBC met the boundary of ICP layer). Note that the red region at the end of the membrane included the preconcentrated hemoglobin which was leaked from the RBCs as hemolysis occurred (Fig. 6(a), *t* = 50 sec).

Unlike the device of *L* = 1000 um, there were no destruction at all in the device of *L* = 6500 um (Fig. 6(b), *t* = 12 sec and 50 sec). Because there existed the bifurcated current path all over the microchannel, stable and non-destructive preconcentration of the RBCs was achieved. The amplification ratio of RBCs were ~40 fold. Note that the steady state length of the ion depletion zone with the applied voltage of 50 V was in the range from 1000 um to 6500 um.

In terms of the current path, the schematic diagrams of developed ion depletion zone at *t* = 50 sec for each cases were depicted in Fig. 6(c) and (d), respectively. In the diagrams, equivalent electrical resistance with different size representing different resistance were also described. The color of major current path was indicated in red. As the ion depletion zone escaped from the protruded membrane, the resistance of the ion depletion zone outside the nanoporous membrane (*R*_*dep1*_) became the largest resistance along the current path. Also the *R*_*dep1*_ was the main factor of the destruction of cellular species during preconcentration process. It induced the amplified electric field and chaotic motions, leading to high shear stress on the species. On the other hand, there was no such *R*_*dep1*_ in Fig. 6(d). In other words, there was minimal amplified electric field and chaotic motions due to the bifurcated current path. As a result, minimal shear stress was applied on the species enabling a stable preconcentration without destruction.

## Conclusions

The research of ICP has focused on unveiling the fundamental electrokinetic processes near a perm-selective membrane. Also, various engineering endeavors recently conveyed several innovative applications using these processes. In this work, the protruded membrane was fabricated in the micro/nanofluidic ICP platform to greatly enhance the surface conduction of the ionic current which is the essential key of suppressing the unwanted instability and high shear stress due to the amplified electrokinetic responses. Experimental visualizations revealed that the ICP layer was initiated from either the end of protruded membrane or the end of microchannel at the dilute limit or the condensed limit, respectively. Current-time measurements and numerical simulations were carried out to verify that the effect of conductance ratio of the electrolyte to the membrane. Consequently, the strong electrokinetic flow associated with the amplified electric field inside ICP layer were significantly suppressed over the protruded membrane at the condensed limit. Using the protruded device, we successfully demonstrated a non-destructive micro/nanofluidic preconcentrator of RBCs. Conclusively, promoting the surface conduction by coating of additional conductive material would successfully suppress the unwanted instability so that the presenting protruded device would be utilized as an effective mean for stabilizing the ICP process even at 1 M concentration and regulating the inherently amplified electrokinetic flow inside ICP layer.

## Electronic supplementary material

Supporting information

Supporting video 1

Supporting video 2
