# Supplementary material for: Ion Concentration Polarization by Bifurcated Current Path
Source: Sci Rep. 2017 Jul 11;7:5091. doi: 10.1038/s41598-017-04646-0 (PMC5505964; doi:10.1038/s41598-017-04646-0)
Supplement: Supplementary file 1 — Supporting information [file 41598_2017_4646_MOESM1_ESM.pdf]

Supporting Information for

# Ion Concentration Polarization by Bifurcated Current Path

Junsuk Kim, Inhee Cho, Hyomin Lee and Sung Jae Kim

**The conductance measurement between a nanoporous membrane and the electrolyte solution inside a microchannel.**

Two devices were fabricated for the measurement of the conductance between a nanoporous membrane and the electrolyte solution inside a straight microchannel. The first device was a simple straight microchannel with the geometries of 15 μm depth, 200 μm width, and 15 mm length (SI Figure 1(a)). The second one had a patterned nanoporous membrane at the bottom of the first device with ~1 μm height of Nafion nanoporous membrane (SI Figure 1(b)). The conductance of the second device would be the sum of microchannel and nanoporous membrane since the nanoporous membrane was paralleled with the microchannel.

The microchannel was filled and flushed with target concentration electrolytes for ~2.5 hours so that the impurities inside the microchannel to be eliminated and the nanoporous membrane to become equilibrium state. The voltage was stepped from -0.1 V to +0.1 V at the rate of 0.05 V / 60 s, where time current transients were saturated. The conductance of each devices with different electrolyte concentrations were determined by obtaining the fitting curve's slope (ionic current vs. applied voltage). Each measurements was repeated at least 5 times with 5 devices for reliability.

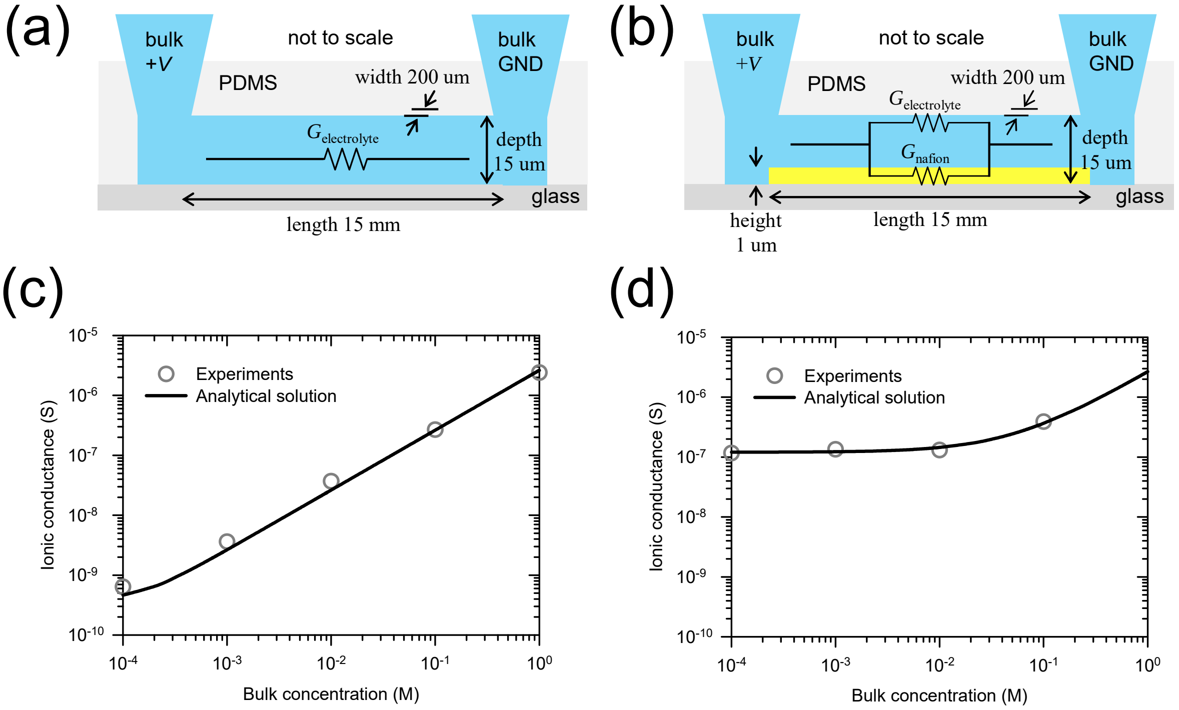


SI Figure 1. Side schematic view of (a) simple straight microchannel device and (b) the device with patterned nanoporous membrane at the bottom. The measured ionic conductance of (c) first and (d) second device with analytical solution.

The ionic conductance of the first device, which only had a microchannel part, was proportional to the bulk concentration (SI Figure 1(c)), while the ionic conductance of the second device, which had a parallel connection of the microchannel and the nanoporous membrane, formed a plateau below a threshold concentration (SI Figure 1(d)). The ionic conductance ($G$) was defined as

$$G = \frac{FA}{L}\sum_i \mu_i N_i \tag{1}$$

where $F$ is the Faraday constant, $A$ is the cross-sectional area, $L$ is the length of the current path, $\mu_i$ is the electrophoretic mobility of $i$-th charge carrier and $N_i$ is the concentration of $i$-th charge

carrier. In an electrohydrodynamic system, the $N_i$ is the Donnan concentration on the cross-section. The Donnan concentration was represented by

$$N_+ = \frac{1}{2}\left(\sqrt{4c_0^2 + N_w^2} + N_w\right) \tag{2}$$

for cation and

$$N_- = \frac{1}{2}\left(\sqrt{4c_0^2 + N_w^2} - N_w\right) \tag{3}$$

for anion, respectively [1]. In the above, $c_0$ is the bulk concentration and $N_w$ is the surface charge concentration which was defined as

$$N_w = -\frac{2q_s}{Fa} \tag{4}$$

where $q_s$ is the surface charge density and $a$ is the equivalent hydrodynamic radius (2 × area / perimeter) [2]. For the case of SI Figure 1(a), Since $a$ was ~14 um and $q_s$ of the PDMS surface was -7 mC/m$^2$ [3,4], $N_+ \approx N_- \approx c_0/2$ if the $c_0$ is larger than 0.1 mM. Hence, the ionic conductance was proportional to the bulk concentration as shown in SI Figure 1(c) and its functional form was

$$G_{electrolyte} = \frac{FA_m c_0(\mu_+ + \mu_-)}{2L} \tag{5}$$

where $A_m$ is the cross-sectional area of microchannel. On the other hand, the surface charge concentration of the nanoporous membrane was non-negligible compared with the bulk concentration due to the electrical double layer overlap so that the ionic conductance of the nanoporous membrane (Nafion) was

$$G_{nafion} = \frac{FA_n}{2L}\left[\mu_+\left(\sqrt{4c_0^{\ 2} + N_n^{\ 2}} + N_n\right) + \mu_-\left(\sqrt{4c_0^{\ 2} + N_n^{\ 2}} - N_n\right)\right] \tag{6}$$

where $A_n$ is the cross-sectional area and $N_n$ is the surface charge concentration of the Nafion. Using the circuit theory, the ionic conductance in the case of SI Figure 1(b) was represented by

$$G = G_{electrolyte} + G_{nafion}\ . \tag{7}$$

The appearance of plateau is attributed to electric double layer (EDL) overlap phenomenon which becomes severe as the bulk concentration decreases. Since the fixed amount of counter-ions existed inside the nanoporous membrane with the severe EDL overlap, the ionic conductance of the nanoporous membrane was independent from the bulk concentration [5].

The ionic conductance of the nanoporous membrane and the electrolyte were separated from each another using the measured conductance of two devices. Then these data was recalculated for the device with protruded membrane since the device had different dimensions. The recalculated data was shown in Figure 1(d) in main text.

**Descriptions for transport phenomena of ionic species inside nanoporous membrane**

From the theory of the Donnan equilibrium[1], the concentrations of cation and anion in nanoporous membrane were expressed as

$$c_+ = \frac{1}{2}\left(\sqrt{4{c_0}^2 + N^2} + N\right) \tag{8}$$

and

$$c_- = \frac{1}{2}\left(\sqrt{4{c_0}^2 - N^2} + N\right) \tag{9}$$

where $c_+$, $c_-$, $c_0$ and $N$ are the concentrations of cation, anion, bulk and fixed charge of the membrane, respectively. Equation (8) and (9) was valid in 1:1 electrolyte solution. When the ionic strength ($c$) was defined as

$$c = \frac{c_+ + c_-}{2}, \tag{10}$$

the cation and anion concentrations were simply represented as following by combining equation (8) – (10) as

$$c_\pm = c \pm \frac{N}{2}. \tag{11}$$

The Nernst-Planck equations for each ionic species were

$$\frac{\partial c_\pm}{\partial t} = -\nabla \cdot \mathbf{j}_\pm \tag{12}$$

where $t$ is the time and $\mathbf{j}_\pm$ is the ionic flux. The ionic flux can be represented by

$$\mathbf{j}_\pm = -D\nabla c_\pm \mp \frac{FD}{RT} c_\pm \nabla \psi + c_\pm \mathbf{u} \tag{13}$$

when the diffusivity of each ionic species was identical. In equation (13), $D$ is the diffusivity, $F$ is the Faraday constant, $R$ is the gas constant, $T$ is the absolute temperature, $\psi$ is the electric potential and $\mathbf{u}$ is the flow field. As we assumed the low water-permeable membrane, the third term in right hand side of above equation was neglected. Substituting equation (11) into equation (13),

$$\mathbf{j}_\pm = -D\nabla\left(c \pm \frac{N}{2}\right) \mp \frac{FD}{RT}\left(c \pm \frac{N}{2}\right)\nabla \psi \, . \tag{14}$$

Equation (12) would be more simplified by following procedures. The Nernst-Planck equations were changed into

$$\frac{\partial}{\partial t}\left(c_+ + c_-\right) = -\nabla \cdot \left(\mathbf{j}_+ + \mathbf{j}_-\right) \tag{15}$$

and

$$\frac{\partial}{\partial t}\left(c_+ - c_-\right) = -\nabla \cdot \left(\mathbf{j}_+ - \mathbf{j}_-\right) \, . \tag{16}$$

When we used the definitions of averaged ionic flux (*i.e.* $\mathbf{J} = (\mathbf{j}_+ + \mathbf{j}_-) / 2$) and ionic current density (*i.e.* $\mathbf{i} = F(\mathbf{j}_+ - \mathbf{j}_-)$), equation (15) and (16) became

$$\frac{\partial}{\partial t}\left(\frac{c_+ + c_-}{2}\right) = -\nabla \cdot \mathbf{J} \tag{17}$$

and

$$F\frac{\partial}{\partial t}\left(c_{+}-c_{-}\right)=-\nabla\cdot\mathbf{i}\quad.\tag{18}$$

Using equation (11) and (14), equation (17) and (18) became

$$\frac{\partial c}{\partial t}=-\nabla\cdot\left(-D\nabla c-\frac{FD}{RT}\frac{N}{2}\nabla\psi\right)\tag{19}$$

and

$$F\frac{\partial N}{\partial t}=-\nabla\cdot\left(-D\nabla N-\frac{2F^{2}D}{RT}c\nabla\psi\right).\tag{20}$$

Assuming the homogeneous membrane charge (*i.e. N* was constant for entire membrane), the time and spatial derivatives of N should be zero so that the equation (20) was written as

$$0=-\nabla\cdot\left(-\frac{2F^{2}D}{RT}c\nabla\psi\right).\tag{21}$$

In this work, equation (19) and (21) were employed as governing equations to describe the transport phenomena inside the nanoporous membrane.

**Legends for Supporting Videos**

**Video 1**. Propagation of ICP layer at each concentrations

**Video 2**. RBC preconcentration with different length of protruded membrane ($L$)

## Reference


1 Schoch, R. B., Han, J. & Renaud, P. Transport phenomena in nanofluidics. *Reviews of Modern Physics* **80**, 839-883 (2008).

2 Hawkins Cwirko, E. & Carbonell, R. G. Transport of electrolytes in charged pores: Analysis using the method of spatial averaging. *Journal of Colloid and Interface Science* **129**, 513-531, doi:http://dx.doi.org/10.1016/0021-9797(89)90466-9 (1989).

3 Kirby, B. J. & Hasselbrink, E. F. Zeta potential of microfluidic substrates: 1. Theory, experimental techniques, and effects on separations. *ELECTROPHORESIS* **25**, 187-202, doi:10.1002/elps.200305754 (2004).

4 Kirby, B. J. & Hasselbrink, E. F. Zeta potential of microfluidic substrates: 2. Data for polymers. *ELECTROPHORESIS* **25**, 203-213, doi:10.1002/elps.200305755 (2004).

5 Stein, D., Kruithof, M. & Dekker, C. Surface-Charge-Governed Ion Transport in Nanofluidic Channels. *Phys. Rev. Lett.* **93**, 035901 (2004).
